# P-1263. Clinical carbapenem-resistant Klebsiella pneumoniae (CRKP) strains readily develop β-lactam/β-lactamase resistance and are hypervirulent

**DOI:** 10.1093/ofid/ofaf695.1454

**Published:** 2026-01-11

**Authors:** Shaoji Cheng, Cornelius J Clancy, M Hong Nguyen

**Affiliations:** University of Pittsburgh, Pittsburgh, Pennsylvania; University of Pittsburgh, Pittsburgh, Pennsylvania; University of Pittsburgh Medical Center, Pittsburgh, Pennsylvania

## Abstract

**Background:**

Hypermutator strains with DNA mismatch repair (MMR) defects are well-recognized among E. coli but rarely described for other Enterobacterales.

**Figure 1.**
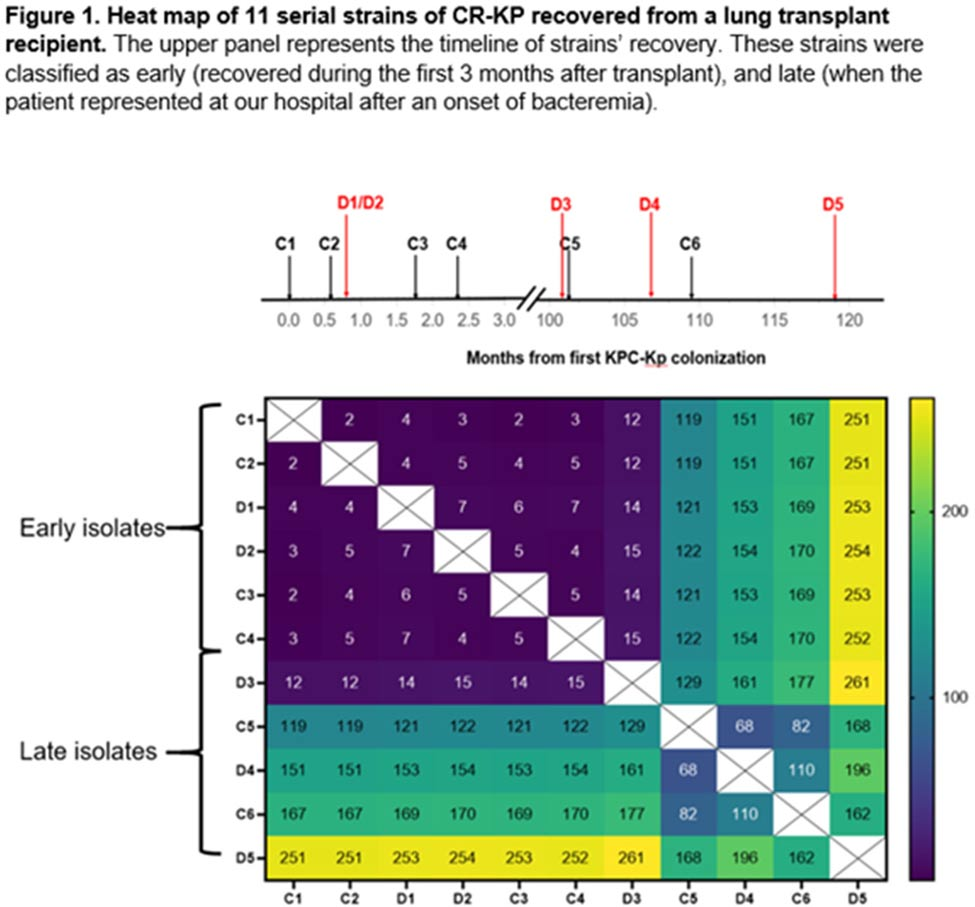
Heat map of 11 serial strains of CRKP recovered from a lung transplant recipient.

**Figure 2.**
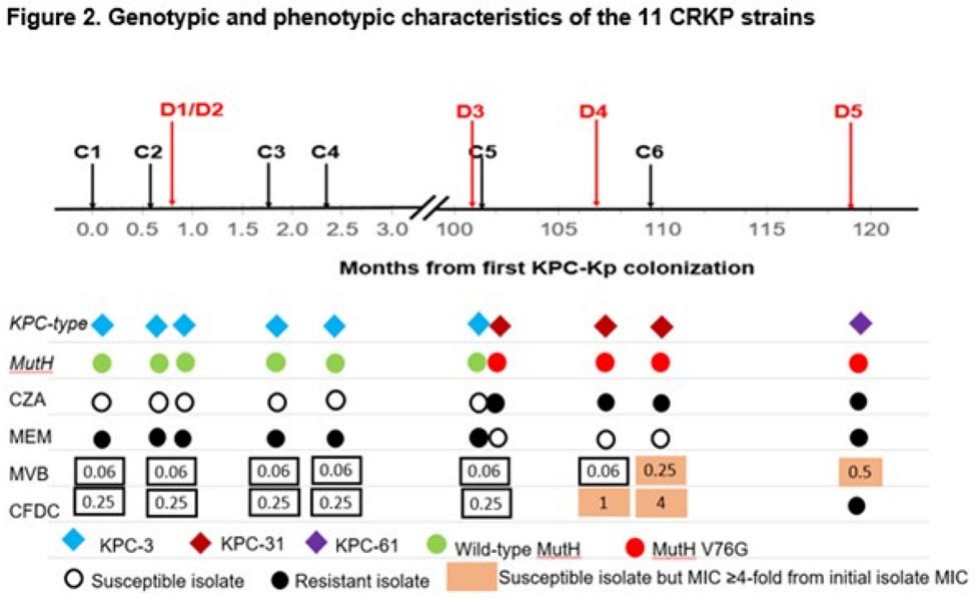
Genotypic and phenotypic characteristics of the 11 CRKP strains

**Methods:**

Eleven CRKP (ST258) strains causing disease (D) or colonization (C) in a lung transplant recipient over ∼4 yrs underwent whole genome sequencing. Clinical and lab-created isogenic mutant strains were tested for hypermutator and other phenotypes.

**Figure 3.**
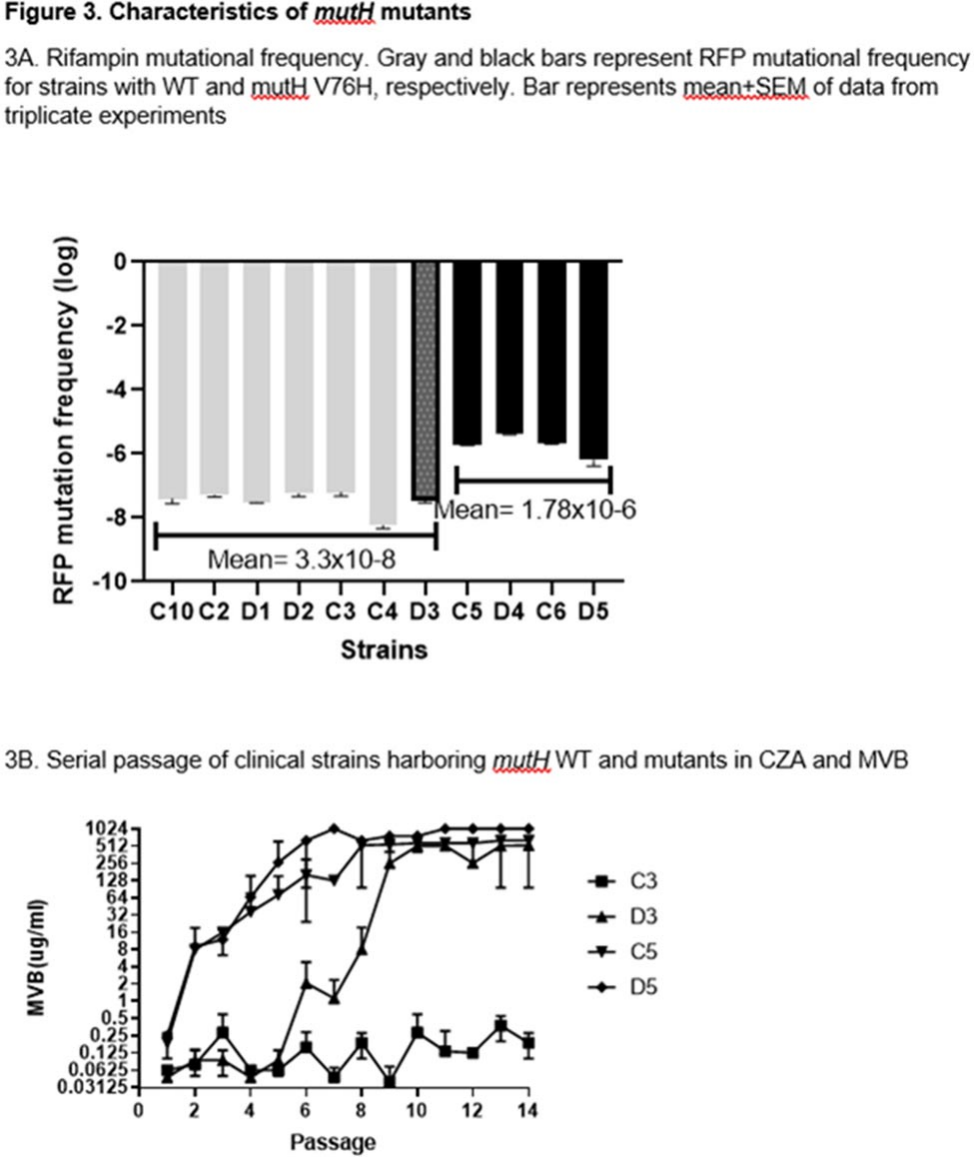
Characteristics of mutH mutants

**Figure 4.**
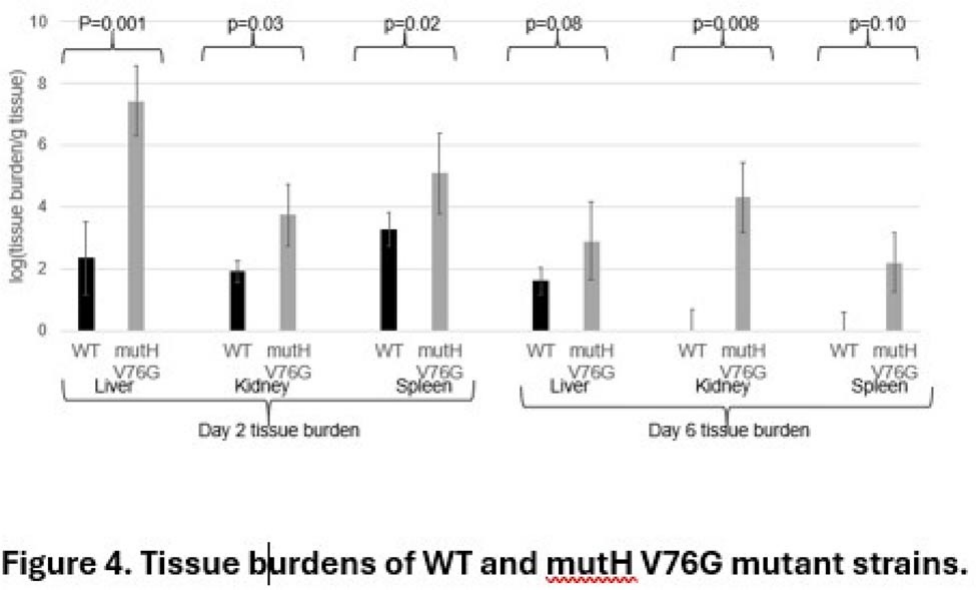
Tissue burdens of WT and mutH V76G mutant strains.

**Results:**

6 early (C1-C4, D1-D2) strains were from 0-2.5 mos post-transplant (Fig 1). 5 late strains were D3 (recurrent bacteremia, 40 mos) and C5, D4, C6 and D5 (41-43 mos). Early strains and D3 were meropenem (MEM)-resistant (R), ceftazidime-avibactam (C/A)-susceptible (S) KPC-3-carriers that differed by 2-7 and 12-15 single nucleotide polymorphisms (SNPs), respectively (Fig 1). C5, D4, C6 and D5 differed from early strains by 119-129, 151-161, 167-177 and 251-261 SNPs, respectively, and carried a mutHV76G (MMR gene) mutation. C5, D4 and C6 were MEM-S, C/A-R KPC-46-carriers (KPC-3-D179Y); D5 was a MEM-R, C/A-R KPC-61-carrier (S171P) (Fig 2). mutHV76G strains had significantly reduced mutH expression (RT-PCR), higher rifampin mutational frequencies (p-values< 0.0001), and greater MEM-vaborbactam (M/V) R during passage in vitro (Fig 3). Isogenic mutHΔ and mutHV76G mutants in D3 had significantly reduced mutH expression, higher rifampin mutational frequencies (p< 0.0001), greater C/A and M/V R during passage in vitro, and enhanced transfer and acceptance of IncX3 plasmid carrying NDM-5 to and from E. coli-074, respectively. In IV mouse infections (5x104 CFU/mouse), isogenic mutHV76G caused higher liver, kidney and spleen burdens than D3 (Fig 4). In mice treated with humanized M/V (0.3 mg/g intraperitoneal Q8hrs, starting 4hrs post-infection), mutHV76G and D3 tissue burdens were each reduced by ≥70%. However, mean mutational frequency (CFU/mL M/V plates/CFU/mL M/V-free plates) of mutHV76G was significantly greater than D3 (3x10-2 vs. < 1x10-8).

**Conclusion:**

Hypermutator CRKP emerged due to mutHV76G, which conferred increased propensity to SNPs, transference and acceptance of antibiotic-R-conferring plasmids, heightened virulence and β-lactam/β-lactamase R in vitro and in vivo. MMR-defective hypermutators merit further investigation among antibiotic-R Enterobacterales.

**Disclosures:**

Cornelius J. Clancy, MD, Merck: Grant/Research Support|Shionogi: Advisor/Consultant M Hong Nguyen, MD, Basilea: Advisor/Consultant|BioMerieux: Grant/Research Support|Melinta: Grant/Research Support|Pulmocide: Advisor/Consultant|Pulmocide: Grant/Research Support

